# Dual redox mediators accelerate the electrochemical kinetics of lithium-sulfur batteries

**DOI:** 10.1038/s41467-020-19070-8

**Published:** 2020-10-15

**Authors:** Fang Liu, Geng Sun, Hao Bin Wu, Gen Chen, Duo Xu, Runwei Mo, Li Shen, Xianyang Li, Shengxiang Ma, Ran Tao, Xinru Li, Xinyi Tan, Bin Xu, Ge Wang, Bruce S. Dunn, Philippe Sautet, Yunfeng Lu

**Affiliations:** 1 Department of Chemical and Biomolecular Engineering, University of California, Los Angeles, CA USA; 2 State Key Laboratory of Supramolecular Structure and Materials, Jilin University, Changchun, China; 3 Department of Materials Science and Engineering, University of Science and Technology Beijing, Beijing, China; 4 Department of Materials Science and Engineering, University of California, Los Angeles, CA USA; 5 Department of Chemistry and Biochemistry, University of California, Los Angeles, CA USA

**Keywords:** Batteries, Electronic structure

## Abstract

The sluggish electrochemical kinetics of sulfur species has impeded the wide adoption of lithium-sulfur battery, which is one of the most promising candidates for next-generation energy storage system. Here, we present the electronic and geometric structures of all possible sulfur species and construct an electronic energy diagram to unveil their reaction pathways in batteries, as well as the molecular origin of their sluggish kinetics. By decoupling the contradictory requirements of accelerating charging and discharging processes, we select two pseudocapacitive oxides as electron-ion source and drain to enable the efficient transport of electron/Li^+^ to and from sulfur intermediates respectively. After incorporating dual oxides, the electrochemical kinetics of sulfur cathode is significantly accelerated. This strategy, which couples a fast-electrochemical reaction with a spontaneous chemical reaction to bypass a slow-electrochemical reaction pathway, offers a solution to accelerate an electrochemical reaction, providing new perspectives for the development of high-energy battery systems.

## Abstract (web summary)

The sluggish electrochemical kinetics of sulfur species remains a major hurdle for the broad adoption of lithium-sulfur batteries. Here, the authors construct an energy diagram of sulfur species to unveil their reaction pathways and propose a general strategy to accelerate electrochemical reactions.

## Introduction

There is an increasing demand for high-energy batteries beyond lithium-ion batteries (LIBs) towards applications such as electric vehicles and drones^1–3^. Sulfur has been considered as one of the most promising candidates owing to its high theoretical energy density, environmental benignity, and low cost^4,5^. When paired with lithium^6–8^ or other anodes^9–11^, the energy density of the full cells can potentially surpass that of LIBs. However, the electrochemical reactions at sulfur cathodes involve multiple polysulfide intermediates with slow reaction kinetics, which results in batteries with low power and energy densities. In addition, outward diffusion of these soluble intermediate species within the cells results in the shuttling effect, deteriorating the capacity retention and shortening the cycling lifetime^12^. Extensive efforts have been made to address these issues, most of which were focused on confining the sulfur species within conductive scaffolds, such as porous carbon particles, graphene, and carbon nanotubes^13,14^. Meanwhile, physical and chemical barriers for the intermediate species were also explored to mitigate the shuttling effect^15–17^. Despite the extensive efforts, fabricating Li–S batteries with high-energy and high-power density remains highly challenging due to the difficulties in determining the critical active species and the reaction pathways at sulfur cathodes, limiting our ability to improve the electrode kinetics.

Based on first-principle calculations, here we construct the electronic energy diagram of various sulfur intermediates to enable a better understanding of the reaction pathways, and the molecular origin of their sluggish electrochemical reaction kinetics. Additionally, we propose a strategy to couple fast electrochemical reactions with spontaneous chemical reactions to circumvent the slow electrochemical reactions of sulfur species. By adding metal oxides as fast-responsive electron-ion reservoirs, which can rapidly react with the sulfur species during charging/discharging process, sulfur cathodes with dramatically improved kinetics, energy, and power density are achieved.

## Results

### Electronic energy diagram of sulfur species

**Fig. 1:**
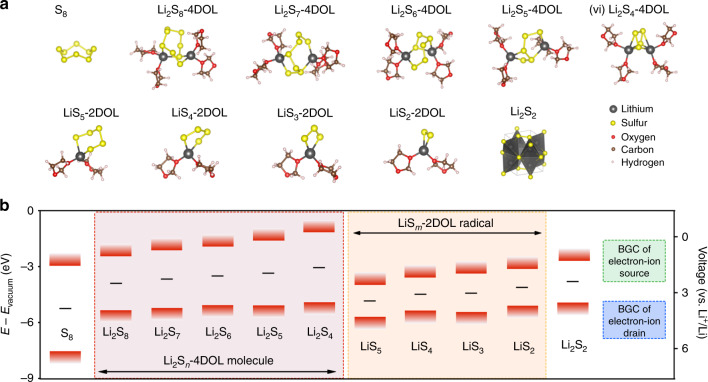
Geometric and electronic structures of sulfur species. **a** The geometric structure of S_8_, Li_2_S_8_-4DOL, Li_2_S_7_-4DOL, Li_2_S_6_-4DOL, Li_2_S_5_-4DOL, Li_2_S_4_-4DOL, LiS_5_-2DOL radical, LiS_4_-2DOL radical, LiS_3_-2DOL radical, LiS_2_-2DOL radical, and Li_2_S_2_ solid. **b** Electronic band edges (highest occupied and lowest unoccupied levels, red bar) and band gap centers (black lines —) of solvated sulfur species aligned with respect to vacuum energy (using hybrid functional B3LYP).

To provide the theoretical guidance toward the rational engineering of the electronic properties of electrode materials, we investigated the geometric and electronic structures of possible sulfur species using density functional theory (DFT) calculations with the B3LYP hybrid exchange-correlation functional (see Supplementary Note 1)^18–21^. Considering that crystalline α-sulfur is only weakly bonded through van der Waals interactions, isolated S_8_ molecule was used to study the electronic properties of sulfur species at the beginning of discharge. Our calculation suggests that *cyclo*-S_8_ exhibits a crown shape with a *D*_4h_ symmetry^22^ (Fig. 1a).

Additionally, lithium sulfide molecules (Li_2_S_n_, 4 ≤ *n* ≤ 8) with different solvation structures were studied to illustrate their electronic structures. Each lithium cation can serve as a coordination center that complexes with solvent molecules (e.g., 1,3-dioxolane (DOL)) and sulfur chains. Although molecular dynamics (MD) simulation in general is the most appropriate method for the complex interactions between solvent and solvates, it is impractical to launch such time-consuming MD simulations with hybrid functionals. Here, we took an alternative approach and investigated the ensemble of solvated lithium sulfide species with different numbers of explicit solvent molecules, to reveal the general trend of their electronic properties. In the simulations, the first coordination shell of lithium cations is saturated explicitly by 0, 1, 2, or 3 DOL molecules and/or sulfur atoms from the sulfur chains (formula is Li_2_S_n_-*x*DOL: *x* = 0, 2, 3, 4, 6). Additional environmental solvent molecules are taken into account by a polarizable continuum model^23^. We considered different lengths of sulfur chains (Li_2_S_n_: *n* ranges from 4 to 8) to represent different states of charge (SOC). The representative structures are shown in Fig. 1a and Supplementary Table 1. In the case of high solvation level (*x* = 6), the Li_2_S_n_ species appear as chain structures, each lithium cations being coordinated with three oxygen atoms from three DOL molecules and a terminal sulfur atom. With fewer explicit solvation molecules (*x* < 6), the lithium cations may also bind with the middle sulfur atoms to fulfill their preferred high coordination number, forming a ring structure.

Radical intermediates also exist in the electrolytes, evidenced by the electron paramagnetic resonance (EPR) spectrum of a Li_2_S_6_ solution that exhibits a typical *S* = 1/2 EPR signal at 5 K (Supplementary Fig. 1)^24,25^. Four different radicals (LiS_2_, LiS_3_, LiS_4_, and LiS_5_) in different solvation states were used to investigate their dimensionless magnetic moment (*g*-factors) with DFT calculations (see Supplementary Note 2). It was found from the EPR spectrum that the magnetic moments of “Li_2_S_6_” solution are close to the *g*-factors of the LiS_4_ and LiS_3_ radicals regardless of their solvation states (Supplementary Table 2), indicating the presence these radicals in the solution and an exergonic dissociation (or disproportionation) from “Li_2_S_6_” to LiS_4_ and LiS_3_. Structural changes of solvated LiS_m_ (*m* = 2–5) radicals show a very similar trend as that in the Li_2_S_n_ series when the number of DOL molecules increases (Supplementary Table 3). LiS_m_ form a ring structure with fewer DOL molecules (*x* < 3), and it turns into a chain structure with additional DOL molecules, where lithium cation forms a tetrahedral coordination with three oxygen atoms and one sulfur atom.

In addition, Li_2_S_2_ is widely accepted as a primary product at the end of a discharge process or at the beginning of charge process^26^. In this work, we adopted the structure determined by previous global optimization result (see Supplementary Note 3)^27^. As shown in Fig. 1a, the unit cell of Li_2_S_2_ is composed of a tetragonal cell with a *P4*_*2*_*/mnm* symmetry. This structure is formed by LiS_4_ tetrahedrons sharing edges and vertexes, in which sulfur atoms form 4 S–Li ionic bonds and one S–S bond (2.117 Å) with neighboring LiS_4_ tetrahedrons.

Furthermore, we investigated the electronic structures of these sulfur species based on their optimized structures. As shown in Fig. 1b, *Cyclo*-S_8_ shows a band gap of 4.59 eV, in accordance with its low electronic conductivity of 1 × 10^−15^ S cm^−1^. Similarly, closed-shell Li_2_S_n_-4DOL (*n* = 4–8) exhibit large band gaps ranging from 2.91 to 3.76 eV, which increase gradually as sulfur chain gets shorter. This is consistent with the increasing overpotentials observed during discharge in the galvanostatic intermittent titration technique (GITT) tests^28^. In contrast, radical LiS_m_-2DOL species present significantly narrower band gaps (1.72–2.07 eV), indicating these radical species are more electronically conductive comparing to Li_2_S_n_ species. Meanwhile, Li_2_S_2_ presents a band gap of 2.29 eV with the SCAN functional, which is a slightly larger value than that previously found with the PBE functional (1.8 eV)^27^, but smaller than that with the hybrid functional HSE06 (3.04 eV, Supplementary Table 6). We will show later that our derived insights are not influenced by the small differences resulting from different functionals.

The electrochemical processes of a sulfur electrode are accompanied by redox reactions, during which electrons are extracted from (charging) or transferred to (discharging) these sulfur species. To illustrate this electrochemical process, we aligned their electronic band edges (i.e., lowest unoccupied molecular orbital (LUMO) and highest occupied molecular orbital (HOMO) for isolated molecules, or valence band maximum (VBM) and conduction band minimum (CBM) for solids) with respect to vacuum energy. As shown in Fig. 1b, sulfur species possess similar HOMO positions, suggesting they can be oxidized under a similar potential. Consistently, Li–S cells typically show a single anodic peak in their cyclic voltammetry profiles. Nevertheless, these sulfur species exhibit quite different LUMOs. Compared with S_8_ (LUMO: −2.95 eV), the Li_2_S_n_-4DOL species present significantly elevated LUMO positions that are increased with decreasing the length of sulfur-chain, indicating that shorter Li_2_S_n_-4DOL species are more difficult to be reduced. On the contrary, the radicals, which are spontaneously formed in Li_2_S_n_ solutions, show much lower LUMO positions than those Li_2_S_n_-4DOL regardless of the length of their sulfur chain. The comparison of these LUMO positions suggests that LiS_m_-2DOL radicals are easier to be reduced comparing to Li_2_S_n_-4DOL species. Therefore, the continuous generation and consumption of LiS_m_ radicals can provide a faster electrochemical pathway during discharge. In the meantime, considering the known deficiencies of DFT exchange correlation functionals, we also evaluated the band gap centers (BGCs) of sulfur species, which are marked as black lines in Fig. 1b. BGC, which are not sensitive to the choice of functional^29^ and formally correct from DFT calculations^30^, can precisely represent the redox potentials of different species. The BGCs shown in Fig. 1b clearly suggest that LiS_m_-2DOL radicals present lower energies (−4.12 to −4.18 eV) comparing to those of Li_2_S_n_-4DOL (−3.9 to −3.05 eV), suggesting that these radical species can be electrochemically reduced before Li_2_S_n_-4DOL species. This is consistent with the previous insights from our band gap calculations, that radicals play a critical role as the most active sulfur intermediates during the discharging process in lithium sulfur battery.

During an electrochemical reaction, electron transfer must occur between the reactant and the external circuit. The diffusion length for electrons that can tunnel through an insulating material (e.g., the sulfur species) is generally less than 1–2 nm^31,32^. This contradiction suggests that only the sulfur species, which are close enough to a conductive network (e.g., carbon black), can be electrochemically reacted. This argument implies that sulfur and sulfur intermediates are dissolved in the electrolyte, and these solvated species migrate to the neighboring conductive networks, where electron transfer and electrochemical reactions occur. Such a dissolution–diffusion–reaction mechanism is consistent with the observation of Li–S cells, which generally require a high electrolyte to sulfur ratio (E/S) to achieve decent electrochemical performances^33^.

### Pseudocapacitive oxides as electron-ion reservoirs

Towards sulfur cathodes with improved kinetics, it is essential to fabricate the electrodes with electron-ion transport networks to enable more efficient transport of electrons and Li^+^ cations. In this circumstance, adapting conductive scaffolds with high specific surface areas may facilitate the electron transfer. However, increasing the surface area of the scaffolds generally reduces their pore volume and the corresponding mass loading of sulfur. An alternative strategy is to incorporate electron-ion reservoirs within the electrodes, which can dynamically and rapidly store and release electrons and Li^+^ cations, while spontaneously react with the sulfur species along the electrochemical process. More specifically, such electron-ion reservoirs should be able to provide electrons and Li^+^ cations to the sulfur species during discharging (serving as an electron-ion source during sulfur reduction). Meanwhile, such reservoirs should be able to accept electrons and Li^+^ cations from the sulfur species during charging (serving as an electron-ion drain during sulfur oxidation).

We hypothesize that electron-ion reservoirs, which are similar to the biochemical mediators (such as NADP^+^/NADPH (NADP^+^: nicotinamide adenine dinucleotide phosphate)), can be used to improve the reaction kinetics of sulfur cathodes. To fulfill the catalytic function, the BGC of electron-ion drain should be lower than the BGCs of sulfur radicals (i.e., −4.84 eV) to accelerate the charging; while the BGC of electron-ion source should be higher than the BGCs of sulfur radicals (i.e., −4.12 eV) to accelerate the discharging. Considering that the redox potential of the sulfur intermediates lies between 2.1–2.4 V (vs. Li/Li^+^), the ideal redox potential for electron-ion reservoirs should be lower than 2.1 V and higher than 2.4 V (vs. Li/Li^+^), respectively. In addition, such electron-ion reservoirs should possess the capability to store and release electrons and Li^+^ cations rapidly and dynamically. Based on these criteria, two pseudocapacitive oxides with fast electrochemical kinetics and long cycling lifetime, orthorhombic Nb_2_O_5_ and birnessite MnO_2_, were employed as representative electron-ion reservoirs in this work. Electrochemically, Nb_2_O_5_/Li_*x*_Nb_2_O_5_ (0 < *x* < 1.25) delivers the majority of its capacity between 1.2 and 2.0 V (vs. Li/Li^+^)^34,35^, while MnO_2_/Li_y_MnO_2_ (0 < *y* < 1) is electrochemically active between 2.4 and 3.6 V (vs. Li/Li^+^)^36,37^. Given that the redox potentials of Nb_2_O_5_ and MnO_2_ fall in the required ranges, they are employed as an electron-ion source and drain, respectively.

**Fig. 2:**
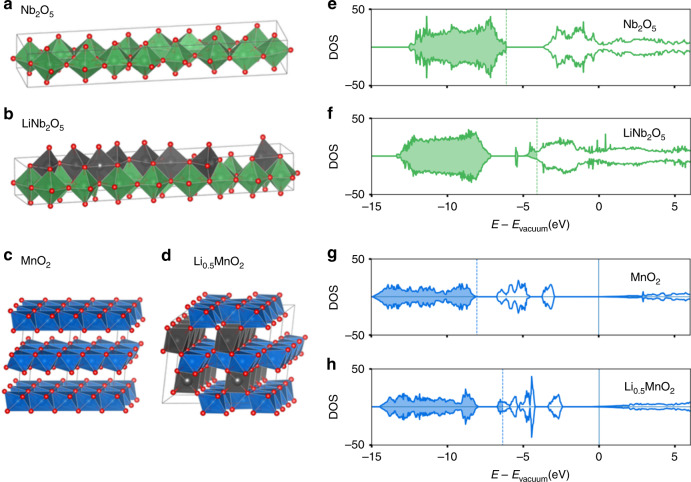
Geometric and electronic structures of oxides. The geometric structure of **a** Nb_2_O_5_, **b** LiNb_2_O_5_, **c** MnO_2_, and **d** Li_0.5_MnO_2_. The corresponding calculated electronic density of states of **e** Nb_2_O_5_, **f** LiNb_2_O_5_, **g** MnO_2_, and **h** Li_0.5_MnO_2_. Lithium, niobium, manganese, and oxygen atoms are shown in gray, green, blue, and red, respectively. Shadowed area represents filled valence band, while unfilled area indicates empty conduction band. The density of states are evaluated by SCAN functional.

Using first-principle calculations, we investigated the geometric structures of Nb_2_O_5_ and MnO_2_ before and after lithiation (see Supplementary Note 4 and 5). As shown in Fig. 2a, orthorhombic Nb_2_O_5_ presents a unit cell with NbO_6_ octahedra and NbO_7_ pentagonal bipyramids connecting each other by sharing edges or vertexes^38,39^ All the niobium cations are stacked layer by layer in the [001] direction forming a layered structure and the interlayer space is serving as fast Li^+^ transport channel and storage space^38^. After lithiation, lithium cations occupy the interstices of NbO_x_ polyhedrons (Fig. 2b), and the structure only experiences a slight expansion (Supplementary Table 4). The unit cell of birnessite MnO_2_ is in a hexagonal structure with *P*_63_/*mmc* symmetry (Fig. 2c)^40,41^. The Mn^4+^ cations are located in MnO_6_ octahedra that share edges within the same layer. The interlayer distance is rather large (4.75 Å), indicating a weak interaction between the layers. After lithiation, lithium cations selectively located in the interlayer region in a small 2 × 2 super cell. The Li cations stay at the center of the distorted octahedral site which is formed by three oxygen anions in the upper layer of MnO_6_ and three in the lower layer of MnO_6_ (Fig. 2d). LiO_6_ octahedra aligns in the [100] direction and no face-sharing structure are formed between LiO_6_ octahedron and MnO_6_ octahedron (Supplementary Table 5).

Figure 2e–h illustrate the density of states (DOS) for the oxides. Nb_2_O_5_ (Fig. 2e) and MnO_2_ (Fig. 2g) present moderate band gaps. Calculated values can be functional dependent, with 2.36 eV (resp. 2.55 eV) for Nb_2_O_5_ and 1.2 eV (resp. 2.95 eV) for MnO_2_ with the SCAN (resp. the HSE06) functional. BGC values are however much less sensitive to the functional choice, with differences smaller than 0.2 eV. The DOS shows that, more importantly, the lithiation process barely changes the shape of their valence bands, but significantly lowers their absolute band energies or elevates fermi energies (Fig. 2f, h). This is related with the electrostatic interaction between lithium 2*s* and oxygen 2*p* orbitals that also enables fast ionic transport within the structure. Given that their conduction bands are partially filled after lithium insertion, LiNb_2_O_5_ and Li_0.5_MnO_2_ are expected to exhibit high electronic conductivity due to their metallic characteristics. The minimum structural distortion during lithiation and delithiation, as well as the high electronic conductivities of Nb_2_O_5_ and MnO_2_ also facilitate fast electrochemical response.

### Electron-ion reservoirs mediated electrochemical reactions

**Fig. 3:**
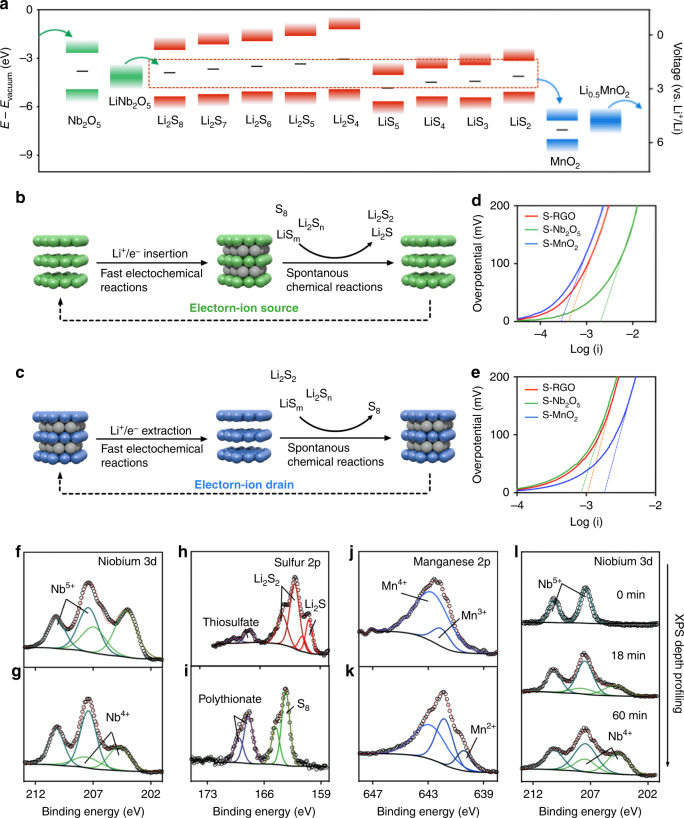
Electron transfers between oxides and sulfur species. **a** Electron transfer pathways at the sulfur cathode with the presence of electron-ion reservoirs. Li_2_S_n_ molecules and LiS_m_ radicals are in solvated states. Schematic illustration of electrochemical and chemical reaction coupling strategy to enhance electrochemical kinetics during **b** discharging and **c** charging process. Tafel plot of RGO, Nb_2_O_5_, MnO_2_ composites in 50 mM Li_2_S_6_ catholyte during **d** cathodic scan and **e** anodic scan. Niobium 3*d* spectrum of **f** LiNb_2_O_5_ and **g** LiNb_2_O_5_–Li_2_S_6_ composites. Sulfur 2*p* spectrum of **h** LiNb_2_O_5_–Li_2_S_6_ composites and **i** MnO_2_–Li_2_S_6_ composites. Manganese 2*p* spectrum of **j** MnO_2_ and **k** MnO_2_–Li_2_S_6_ composites. **l** XPS depth profiling of Nb_2_O_5_ electrode in 50 mM Li_2_S_6_ catholyte at 2.4 V vs. (Li/Li^+^) during discharging.

Figure 3a shows the electronic band edges and BGCs of the sulfur species and the oxides with respect to vacuum energy. During the discharging of sulfur electrodes (reduction of the sulfur intermediates), electrons and Li^+^ are inserted into Nb_2_O_5_ converting it to LiNb_2_O_5_, generating an electron-ion source. Subsequently, as-stored electrons and Li^+^ in the LiNb_2_O_5_ can be transferred to sulfur species (e.g., LiS_m_ radicals) spontaneously, facilitating their reduction reactions. Meanwhile, LiNb_2_O_5_ is converted back to Nb_2_O_5_, which can be regenerated after accepting electrons and Li^+^ from the external circuit and electrolyte, respectively. Given that Li_2_S_n_ molecules can be spontaneously converted into LiS_m_ radicals (Supplementary Fig. 1) with lower BGCs (stronger oxidizing agents), the continuous reaction between LiS_m_ radicals and Nb_2_O_5_/LiNb_2_O_5_ prompts the conversion from Li_2_S_n_ to Li_2_S_2_/Li_2_S. This process can also be explained from an atomic-scale perspective. As the driving force of electrochemical process, electron transfer process is always accompanied by subsequent transport of Li^+^ to the same location/species. For example, during discharging, the reduced sulfide species (Li_2_S_n_/LiS_m_ with an extra electron) have to complex with Li^+^ in a fast manner to minimize the drop of cell potential. As a supercapacitor material, Nb_2_O_5_/LiNb_2_O_5_ can store significantly more electron/Li^+^ (160 mAh g^−1^ under a current density of 20 mA g^−1^, corresponding to 0.1C rate)^42^ compared to that of carbon (5 mAh g^−1^ under a current density of 20 mA g^−1^)^43^, and in particular fast Li^+^ transport can be realized without significant change in electrochemical potential. Therefore, Nb_2_O_5_/LiNb_2_O_5_ species can stabilize the reduced sulfur species locally and timely, resulting in an efficient discharging interface. In contrast, carbon particles are less efficient in stabilizing the reduced sulfide species and require large overpotential to drive this electrochemical process. In the meantime, sulfur intermediates bind stronger with Nb_2_O_5_/LiNb_2_O_5_ as evidenced on Fig. 3h, which also prompts the electron transfer process.

Similarly, during the charging process (oxidation of the sulfur intermediates), electrons and Li^+^ are extracted from Li_0.5_MnO_2_, forming an electron-ion drain MnO_2_. As shown in Fig. 3a, the BGC of MnO_2_ is lower than those of the sulfur intermediates, allowing the flow of the electrons from Li_2_S_n_/LiS_m_ to MnO_2_. The continuous regeneration of MnO_2_ from Li_0.5_MnO_2_, and the spontaneous reaction between sulfur species and MnO_2_ drive the conversion from Li_2_S_n_/LiS_m_ to S_8_. From the atomic-scale perspective, the presence of Li_0.5_MnO_2_/MnO_2_ enables the efficient migration of Li^+^ away from the oxidized sulfide species (Li_2_S_n_/LiS_m_ with an electron hole), ensuring the continuity of the charging process even under high current densities.

Linear voltammetry was employed to evaluate the conversion rate of sulfur intermediates in the presence of different oxides. We synthesized orthorhombic Nb_2_O_5_ and birnessite MnO_2_ nanoparticles on reduced graphene oxide (RGO) using a hydrothermal method. As shown in Supplementary Fig. 2, these crystalline nanoparticles are homogenously grown on the RGO sheets with a weight percentage around 10 wt% (Supplementary Fig. 3). These carbon/oxide composites were mixed with polymeric binders and carbon black to form electrodes, which were subjected to linear voltammetry with Li_2_S_6_ catholyte. Figure 3d, e present the Tafel plots of the electrodes of RGO, Nb_2_O_5_/RGO, and MnO_2_/RGO. These plots deviate sharply from a linear behavior as the overpotential (*η*) approaches to zero, while the linear segments are extrapolated to an interception of log *i*_0_. According to the Butler–Volmer model, the standard rate constant (*k*^0^) of an electrochemical reaction is proportional to its exchange current (*i*_0_). As shown in Fig. 3d, the Nb_2_O_5_/RGO electrode exhibits a much higher *i*_0_ in comparison with that of the RGO electrode (2 vs. 0.41 mA). During anodic scans, the *i*_0_ of the RGO, Nb_2_O_5_/RGO or MnO_2_/RGO electrodes is 1.0 mA, 0.85 mA, and 1.82 mA, respectively, suggesting that the oxidative kinetics for the MnO_2_/RGO electrode is 82% faster than that of the RGO electrode (Fig. 3e). Collectively, these studies confirm Nb_2_O_5_/LiNb_2_O_5_ and MnO_2_/Li_0.5_MnO_2_ can be used as effective electron-ion reservoirs/drains for sulfur cathodes.

On the other hand, CV scanning tests can reveal the difference in the apparent diffusion coefficient of lithium ions *D*(Li^+^) in the electrodes. Here, we used these carbon/oxide composites as the carbon hosts and synthesized sulfur composites with a sulfur loading of 80 wt% (Supplementary Fig. 4), and further fabricated electrodes using slurry casting method (Supplementary Fig. 5). Given that the weight percentages of oxide nanoparticles in the carbon/sulfur composite are around 2 wt%, such a small amount does not significanltly change the charge transfer resistence or the ionic conductivity of the cathodes (Supplementary Fig. 6). We investigated the electrochemical responses of sulfur cathodes under different sweep rates ranging from 0.1 to 0.5 mV s^–1^ (Supplementary Fig. 7). These cathodes exhibit two cathodic peaks, which can be attributed to the reduction of S_8_ to sulfur intermediates ($i_{\mathrm{p}}^{{\mathrm{c}},1}$) and their subsequent reduction to Li_2_S_2_/Li_2_S ($i_{\mathrm{p}}^{{\mathrm{c}},2}$). During the anodic sweep, there is one peak resulting from the conversion of Li_2_S_2_/Li_2_S to sulfur intermediates and S_8_ ($i_{\mathrm{p}}^{\mathrm{a}}$). All three cathodes exhibit a linear relationship between cathodic/anodic peak currents (*I*_p_) and the square root of sweep rates (*ν*), indicating a diffusion-limited process (Supplementary Fig. 7b–d). According to classical Randles–Sevcik equation, the slope of the curve (*I*_p_/*ν*^0.5^) correlates to the diffusion coefficient of lithium ions *D*(Li^+^) of the corresponding electrochemical step. Supplementary Fig. 7e compares the relative *D*(Li^+^) of three sulfur composites normalized by that of S–RGO. During the cathodic sweep, both Nb_2_O_5_ and MnO_2_ promote the conversion from S_8_ to Li_2_S_n_, whereas only Nb_2_O_5_ assists the formation of Li_2_S_2_/Li_2_S. On the other hand, the mediation effect of MnO_2_ is more pronounced on charge, facilitating the oxidation of sulfur species to S_8_. The conclusion from the CV scanning test is consistent with that from the Tafel plot.

Experimentally, the electron transfer between LiNb_2_O_5_, MnO_2_ and the sulfur species was demonstrated using Li_2_S_6_ solution as a representative. Upon mixing with LiNb_2_O_5_, the color of the Li_2_S_6_ solution changed from brown to yellow (Supplementary Fig. 8). The solid product was then separated from the solution and analyzed with XPS. As shown in Fig. 3f, g, the content of Nb^4+^ (core level shift (CLS) 203.4, 206.1 eV)^44,45^ in LiNb_2_O_5_ is decreased from 48.8 to 30% after the reaction, indicating that LiNb_2_O_5_ was oxidized. Correspondingly, the sulfur 2*p* spectrum illustrates the reduction of Li_2_S_6_ with the formation of Li_2_S_2_ (CLS 161.7 eV) and Li_2_S (CLS 160 eV)^46,47^ (Fig. 3h). Besides, a small amount of thiosulfate group ([S_2_O_3_]^2−^, CLS 166.8 eV)^17,47^ is formed on the surface, which could serve as the active site for electron transfers. A similar electron transfer process was also observed between Li_2_S_6_ and MnO_2_, which is mainly composed of Mn^4+^ (CLS 643 eV)^48^ (Fig. 3j). Upon the addition of MnO_2_, the Li_2_S_6_ solution changes to colorless (Supplementary Fig. 8). XPS studies suggest that Mn^4+^ is reduced to Mn^3+^ (CLS 642 eV) and Mn^2+^ (CLS 640.2 eV) (Fig. 3k), while Li_2_S_6_ is oxidized to S_8_ (CLS 163.3 eV)^46,47^ (Fig. 3i). Meanwhile, polythionate group (CLS 167.9 eV), which is composed of thiosulfate groups and sulfur chain, is also generated on the surface of MnO_2_. These experiments imply that, when the band edges of the metal oxides are properly aligned with the band structures of active sulfur species (radicals in this case), chemical reactions can occur spontaneously between the metal oxide and the sulfur species. Such chemical reactions circumvent the slow electrochemical pathway between the sulfur species and carbon electrode, leading to improved sulfur reaction kinetics.

Thermodynamically, the redox reaction of Nb_2_O_5_ mainly occurs between 1.2 and 2.0 V vs. Li/Li^+^; one may be concerned that LiNb_2_O_5_ can be not generated at the discharge voltage of sulfur cathodes (2.1–2.4 V vs. Li/Li^+^). In terms of electrochemical kinetics, Nb_2_O_5_ is a pseudocapacitive oxide with extremely fast kinetics (e.g., ~70% of capacity retention with increasing the current density from 0.2 to 20 A g^−1^), especially when compared with sluggish battery materials. During the discharging process, particularly under a high current density, it is possible that Nb_2_O_5_ within sulfur cathodes is preferably lithiated, forming LiNb_2_O_5_ with a transiting local voltage lower than 2.0 V. Subsequent reaction of the LiNb_2_O_5_ with the sulfur species, followed by regeneration of LiNb_2_O_5_, constructs a mediated electrochemical reaction with accelerated electrochemical kinetics.

This effect was confirmed by ex situ XPS analysis, where 0.5 M LiTFSI and 50 mM Li_2_S_6_ solution was used as the catholyte, and Nb_2_O_5_ electrode and lithium foil were employed as the working and counter electrode, respectively. The cell was scanned at a constant sweep rate of 5 mV s^–1^, during which the sweeping was stopped at 2.4 V vs. Li/Li^+^ and the electrode was subject to an XPS analysis. Figure 3l show the XPS spectra of the Nb_2_O_5_ electrode before and after etching for 18 min, and 60 min using an Argon-ion gun, respectively. As expected, increasing amount of Nb^4+^ was found towards the current collector from the liquid-electrode interface. This observation confirms that Nb_2_O_5_ can be effectively converted to LiNb_2_O_5_ even at an electrode voltage higher than 2.0 V. The increasing content of Nb^4+^ towards the current collector is consistent with the reaction of the sulfur species with LiNb_2_O_5_, as well as diffusion of the sulfur species to the interior of the electrode.

### Electrochemical performance of sulfur cathodes with electron-ion reservoirs

The energy density of lithium-sulfur battery is closely related to the mass loading of sulfur in cathode and the ratio between electrolyte to sulfur (*E*/*S*). Although Nb_2_O_5_ and MnO_2_ have been incorporated into sulfur cathodes before, they have not been evaluated side-by-side in thick sulfur electrodes with precisely control amount of electrolyte. The electrochemical behaviors obtained from previous thin electrodes can’t be readily transformed into that of thick electrodes in practical applications. Herein, we used thick sulfur cathodes (areal mass loading of 7 mg cm^−2^) with an *E*/*S* ratio of 7 in coin cell as a proof-of-concept demonstration. The fast conversion of sulfur intermediates is expected to suppress the outward dissolution of polysulfides and enhance capacity retention.

**Fig. 4:**
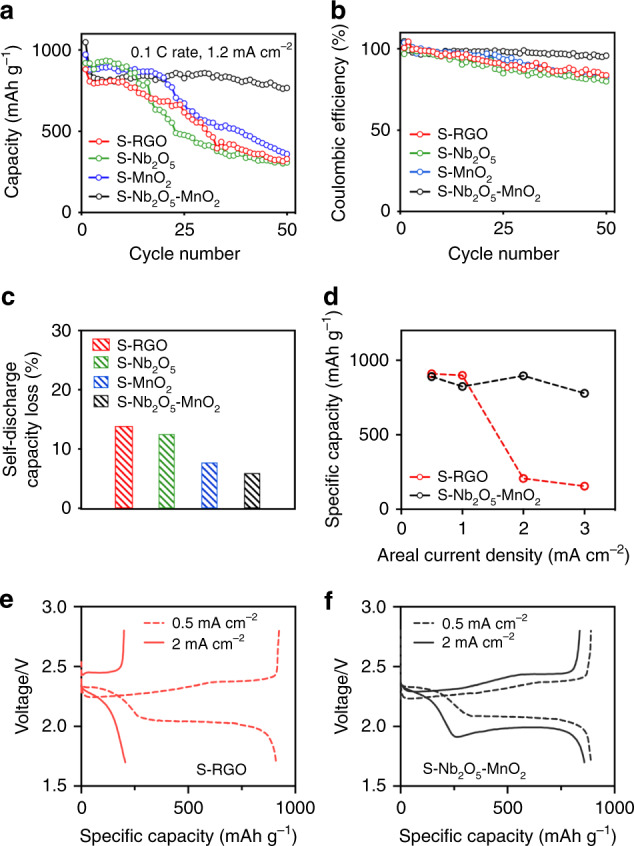
Electrochemical performance of lithium–sulfur batteries with/without oxides. **a, b** Galvanostatic cycling performance of sulfur electrodes under a current density of 167.5 mA g^−1^ (0.1C rate). **c** Self-discharge capacity loss of sulfur electrodes after resting for 24 h at 2.1 V (vs. Li^+^/Li) during the 5^th^ cycle. **d** Specific capacities of S–RGO and S–Nb_2_O_5_–MnO_2_ electrodes under various current densities. **e** Representative voltage–capacity profiles of S–RGO electrode and **f** S–Nb_2_O_5_–MnO_2_ electrode under current densities of 0.5 and 2.0 mA cm^−2^.

Figure 4a shows the specific capacity of the sulfur electrodes with different oxides under a current density of 1.67 mA g^−1^, corresponding to 0.1C rate. After 50 cycles, the S–Nb_2_O_5_–MnO_2_ electrode still delivers a reversible capacity of 767.2 mAh g^−1^, whereas the S–RGO electrode experiences a fast capacity decay after ten cycles and maintains a low capacity of 329.9 mAh g^−1^ at the 50^th^ cycles. Consistently with the significant improvement in cycling stability, the S–Nb_2_O_5_–MnO_2_ electrode shows significantly improved Coulombic efficiency (Fig. 4b). For comparison, we also evaluated the cycling stability of sulfur electrodes with Nb_2_O_5_ or MnO_2_ under the same testing conditions. Although S–Nb_2_O_5_ and S–MnO_2_ electrodes present slightly enhanced specific capacities comparing to that of S–RGO electrode during the initial ten cycles, they only maintained marginal improvements after 50 cycles (S–RGO: 329.9 mAh g^−1^, S–Nb_2_O_5_: 307.6 mAh g^−1^, S–MnO_2_: 359.8 mAh g^−1^). The capacity decay observed in S–Nb_2_O_5_ (or S–MnO_2_) electrodes can be attributed to the inefficient utilization of sulfur species, during the subsequent charging (or discharging) process. The slow conversion of sulfur intermediates leads to their accumulation in the electrolyte, prompts their side-reactions with lithium metal anode, and results in the drop of Coulombic efficiency. This comparison further demonstrated the importance of having both Nb_2_O_5_/LiNb_2_O_5_ and Li_0.5_MnO_2_/MnO_2_ as electron-ion reservoirs in thick sulfur electrodes to accelerate both discharging and charging processes. In addition, we want to point it out that incorporating oxides in thin sulfur electrodes where the transport of electrons/Li^+^ is sufficiently fast, the improvement in electrochemical performance can be marginal or even negligible (Supplementary Fig. 9).

Meanwhile, the efficient conversion of sulfur intermediates also minimizes the outward diffusion of sulfur species, and mitigates resulted self-discharging capacity loss (Fig. 4c). Newly assembled Li–S cells were cycled at 0.05C rate (1C = 1675 mA g^−1^) for the first cycle and then at 0.1C rate for three cycles. During the 5^th^ cycle, the discharging process was paused at 2.1 V (vs. Li^+^/Li) where the generation of soluble lithium polysulfides is maximum. After resting for 24 h, the discharging process is resumed. Here, we define the self-discharge capacity loss as (*C*_4_ − *C*_5_)/*C*_4_ × 100% where *C*_n_ represent the discharge capacity during the *n*^th^ cycle. The capacity loss observed in S–Nb_2_O_5_–MnO_2_ electrode is the lowest among all four electrodes (S–RGO: 13.88%, S–Nb_2_O_5_: 12.53%, S–MnO_2_: 7.7%, S–Nb_2_O_5_–MnO_2_: 5.93%), re-illustrating the necessity of having both oxides in the cathode. In addition, no obvious polysulfide shuttling or decrease in CE (Supplementary Fig. 10) were observed during the subsequent charging process in all four Li–S cells. Different from the irreversible capacity loss observed in thin electrodes, the majority of the capacity loss in thick electrodes can be recovered in the next cycle (Supplementary Fig. 11), which may be attributed to limited amount of electrolyte and the associated high viscosity.

To explore the potential applications of S–Nb_2_O_5_–MnO_2_ electrodes under high power demands, we evaluated their electrochemical behaviors under various current densities ranging from 0.5 to 3 mA cm^−2^ (Fig. 4d). To avoid the influence from the fast degradation of lithium metal anodes under such harsh conditions, we choose to run the galvanostatic test under each current density for only one cycle instead of multiple cycles. S–Nb_2_O_5_–MnO_2_ electrode exhibits excellent electrochemical behaviors by delivering comparable specific capacities under such large range of current densities. S–RGO electrode, which delivers a similar specific capacity under a small current density of 0.5 mA cm^−2^, presents completely different electrochemical response under high current densities. When the current density is increased to 2 mA cm^−2^, the capacity from the second discharging plateau of sulfur cannot be utilized, resulting in a pronounced drop of available capacity (206.4 mAh g^−1^) in S–RGO electrode (Fig. 4e). This result is consistent with the sluggish transport of electrons from carbon matrix and Li^+^ from the limited electrolyte in sulfur electrodes. In contrast, with the assistance of Nb_2_O_5_/Li_x_Nb_2_O_5_ (electron-ion source) and MnO_2_/Li_y_MnO_2_ (electron-ion drain), the transport electrons/Li^+^ to and from sulfur species can be dramatically accelerated, leading to efficient utilization of active materials even under fast discharging and charging conditions (Fig. 4f). These studies collectively confirm a dramatically improved electrochemical kinetics upon the addition of the dual oxides in thick sulfur cathodes.

In addition, the advantage of using dual redox mediators becomes more pronounced with lower content of electrolyte (Supplementary Fig. 12a, b). When *E*/*S* ratio is 7, the initial capacity of S–RGO and S–Nb_2_O_5_–MnO_2_ electrodes at 0.05C rate are similar. However, when the *E*/*S* ratio is lowered to 5, the utilization of sulfur in S–RGO significantly decreased with a large voltage drop of the second plateau. In contrast, the electrochemical behavior of S–Nb_2_O_5_–MnO_2_ electrode remains similar disregard of the decreased amount of electrolyte, delivering a much higher energy density on the cell level (Supplementary Fig. 12c). Of note, a number of materials (e.g., oxides^49^, sulfides^50,51^, nitrides^52^, and carbides^53^) have been explored to enhance the redox kinetics of sulfur electrodes as well. Given that the pseudocapacitive Nb_2_O_5_–MnO_2_ dual redox mediators enabled the fast and efficient transport of electron/Li^+^ both to and from sulfur intermediates, it leads to Li–S cells with better rate performance (Supplementary Fig. 13).

## Discussion

In this work, we investigate the electronic structures of all possible sulfur species involved in lithium-sulfur batteries and construct an electronic energy diagram to illustrate their reaction pathways. By decoupling the contradictory requirements for the acceleration of charging and discharging processes, we rationally select two pseudocapacitive oxides (Nb_2_O_5_/Li_x_Nb_2_O_5_ and MnO_2_/Li_y_MnO_2_) as electron-ion reservoirs (source and drain), which can enable the efficient transport of electron/Li^+^ to and from sulfur intermediates respectively. Adapting such electron-ion reservoirs enables the fabrication of sulfur electrodes with fast electrochemical kinetics, leading to enhanced areal capacity and power performance, as well as prolonged cycling life. This strategy that couples a fast electrochemical reaction with a spontaneous chemical reaction to circumvent an sluggish electrochemical reaction can be readily extended to other electrode materials with slow electrochemical kinetics such as silicon and phosphorus, opening a new avenue for lithium batteries and other electrochemical devices.

## Methods

### Synthesis of RGO and RGO–metal oxides composites

Reduced graphene oxide (RGO) was prepared by oxidation of natural graphite flacks (Sigma-Aldrich) following the Hummers method followed by reduction using ascorbic acid (Sigma-Aldrich) at 90 °C for 2 h (pH = 10). The resulted solid product was washed with deionized water for several times until pH reaches 7. After freeze-drying, RGO was obtained.

The RGO–Nb_2_O_5_ composites were synthesized according to the previously reported procedure. Briefly, 25 mg NbCl_5_ (Sigma-Aldrich) was dissolved in 5 mL of ethanol (Fisher Scientific). In a separate vial, 110.7 mg RGO was dispersed in 50 mL ethanol by sonication. Both vials were chilled in ice bath for 2 h. The two solutions were then mixed while 0.5 mL oleylamine (Sigma-Aldrich) and 8.3 µL deionized water were slowly injected. The solution was heated at 75 °C in an oil bath with magnetic stirring for 6 h. The resulted product was washed with ethanol and water to remove excess oleylamine and then freeze-dried. After annealing at 600 °C for 3 h in argon, RGO–Nb_2_O_5_ composites were obtained.

The RGO–MnO_2_ composites were synthesized at room temperature. First, 3.175 mg MnSO_4_ • H_2_O (Sigma–Aldrich) was dissolved in 2.5 mL deionized water. In a separate vial, 109.5 mg RGO was dispersed in 15 mL deionized water by sonication. The two solutions were then mixed and form a homogenous solution. Then, 10 mg KMnO_4_ (Sigma-Aldrich) was dissolved in 2.5 mL deionized water and added to the previous solution. This solution was further stirred at room temperature for 12 h. The resulting solid product was washed with water for several times and then freeze dried.

### Synthesis of S–RGO and S–RGO–metal oxide composites

The sulfur and RGO composites (denoted as S–RGO composites) were prepared using a liquid infiltration method at 159 °C for 4 h. S–Nb_2_O_5_ composites, S–MnO_2_ composites and S–Nb_2_O_5_–MnO_2_ were synthesized via similar method by replacing RGO with RGO–Nb_2_O_5_, RGO–MnO_2_, and mixed RGO–Nb_2_O_5_ and RGO–MnO_2_ composites (weight ratio = 1:1). The weight ratio between sulfur and RGO (or RGO–metal oxides composites) was 4:1.

### Preparation of sulfur cathodes

Sulfur cathodes were prepared using a slurry casting method. Carbon/sulfur composites, carbon fiber (Pyrograf Product Inc.) and sodium alginate (Sigma-Aldrich, 4 wt% solution in deionized water) were mixed with a weight ratio of 8:1:1 to form a homogenous slurry, which was casted onto carbon-coated aluminum foil with a doctor blade. The resulting electrodes were dried at 70 °C in vacuum for 4 h.

### Preparation of Li_2_S_6_ solution

Twenty millimolar of Li_2_S_6_ solution was prepared by mixing stoichiometric amounts of elemental sulfur (Sigma-Aldrich) and Li_2_S (Alfa Aesar) in DOL: DME (Sigma-Aldrich, volume ratio 1:1). A homogenous dark–yellow solution of Li_2_S_6_ was obtained after stirring for 24 h at 130 °C.

### Electrochemical measurements

To evaluate the electrochemical performance, 2032-type coin cells (MTI Corporation) were assembled with polypropylene separator (Celgard 2500), and lithium foil (Alfa Aesar) as the anodes. 0.5 M LiTFSI (Sigma-Aldrich) and 2 wt% LiNO_3_ (Sigma-Aldrich) in DOL/DME was used as electrolyte. Cyclic voltammetry analysis was performed on a Bio-Logic VMP3 electrochemical workstation with a three-electrode configuration. Lithium foils were used as both counter electrode and reference electrode. Linear voltammetry analysis was performed on Solartron 1860/1287 electrochemical interface with two-electrode configuration. 0.5 M LiTFSI and 0.1 M Li_2_S_6_ in DOL/DME solution was used as the catholyte and lithium foil was used as the counter electrode. Galvanostatic charge–discharge measurements were carried out using Land CT2000 battery tester in a voltage range of 1.7–2.8 V for all rates. Specific capacities were calculated with respect to the mass of sulfur.

### Material characterization

XRD measurements were performed on Rigaku MiniFlex instrument using the copper Kα radiation (*λ* = 1.54 Å). TGA was performed on a TA Instrument SDT Q600 employing a heating rate of 5 °C min^–1^ from 40 to 700 °C under airflow. SEM and TEM studies were conducted on a ZEISS Supra 40VP and Titan S/TEM, respectively. For XPS studies, the samples were sealed in a transporter in the Argon-filled glove box before being quickly transferred to the high-vacuum chamber of XPS (AXIS Ultra DLD) for analysis. All the spectra were fitted to Gaussian–Lorentzian functions and a Shirley-type background using CasaXPS software. The binding energy values were all calibrated using C 1s peak at 284.5 eV.

### DFT calculations

The periodic structures including MnO_2_, Li_0.5_MnO_2_, Nb_2_O_5_, LiNb_2_O_5_, and Li_2_S_2_ are calculated with VASP^54–58^. The SCAN functional^59^ is used for describing the exchange–correlation interactions for solid systems. It has been shown that SCAN functional is very accurate for the electronic structure of MnO_2_, alkali intercalated MnO_2_, and a wide range of materials^60–62^. The energy cutoff for plane waves is 400 eV. The density of k-mesh is large enough to make sure that the energy difference is smaller than 0.01 eV/unit cell. HOMO/LUMO positions of isolated molecules including S_8_, LiS_4_, and LiS_3_ radicals are calculated with the Gaussian09 package^63^ at the level of B3LYP functional level with 6–311 + +(d, p) basis sets.

### Alignment of absolute band positions

We used the following scheme to align the band edge positions (including VBM and CBM) of different materials with the vacuum energy:

1 $$E_{\mathrm{i}}\,=\,\left[ {E_{\mathrm{i}}^{{\mathrm{bulk}}}\,-\,E_{{\mathrm{ref}}}^{{\mathrm{bulk}}}} \right]\,+\,\left[ {E_{{\mathrm{ref}}}^{{\mathrm{slab}}}\,-\,E_{{\mathrm{vac}}}^{{\mathrm{slab}}}} \right].$$

The first term calculates the difference between band edge energy *E*_i_ (which is either VBM or CBM) and a reference state *E*_ref_. Here we use the energy of a semicore orbital as the reference state. The second term calculates the difference between the reference state and vacuum energy in the slab model. The reference state is chosen as the semicore orbitals because they are rarely influenced by their chemical environment. In this work, we choose the 4*s* orbital of niobium atom, 3*s* orbital of manganese atom, 1*s* of lithium atom (for Li_2_S_2_ only) as the reference state, respectively. It should be notated that the energy of semicore orbitals can be also influenced by the Madelung potential. If the adopting slab model is too thin, the Madelung potential will be different between bulk and thin slab. Therefore, we ensured the slab model has a sufficient large thickness (larger than 35 Å) in all the calculations to minimize the difference of the Madelung potential at the center of the slab.

## Supplementary information

Supplementary information

## Data Availability

The data that support the plots within this article and other findings in this study are available from the corresponding author upon reasonable request.
