# Supplementary material for: Dual redox mediators accelerate the electrochemical kinetics of lithium-sulfur batteries
Source: Nat Commun. 2020 Oct 15;11:5215. doi: 10.1038/s41467-020-19070-8 (PMC7567085; doi:10.1038/s41467-020-19070-8)
Supplement: Supplementary file 1 — Supplementary information [file 41467_2020_19070_MOESM1_ESM.pdf]

Supplementary Information for

Dual redox mediators accelerate the electrochemical kinetics of lithium-sulfur batteries

Liu et al.

Supplementary Note 1: Calculations on Li_2S_n with different solvation models. The calculations are conducted with Gaussian09 package using 6-311++G(d,p) basis set and B3LYP functional. Environmental solvents are considered with implicit solvation model ($\varepsilon = 4.33$). The band gap center (BGC) is the average of the HOMO and LUMO levels. Structure optimizations of close-shell Li_2S_n are conducted by Gaussian 09 packages in the level of 6-311++ (d,p) basis sets and B3LYP functional.

Supplementary Table 1. Geometric and electronic band structures of Li_2S_n with different solvation models.

Species	HOMO (eV)	LUMO (eV)	BGC (eV)	Band gap (eV)	Structure
DOL	-7.48	-0.15	-3.82	7.33	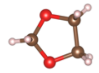
Li_2S_4	-5.68	-1.62	-3.65	4.05	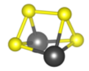
Li_2S_4-2DOL	-5.46	-1.46	-3.46	4.00	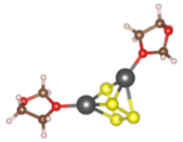
Li_2S_4-3DOL	-5.22	-1.30	-3.26	3.92	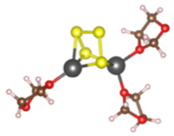
Li_2S_4-4DOL	-4.93	-1.17	-3.05	3.76	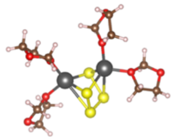
Li_2S_4-6DOL	-4.68	-0.94	-2.81	3.74	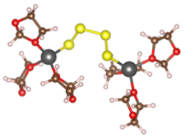
Li_2S_5	-5.58	-1.92	-3.75	3.66	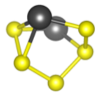

Li_2S_5-2DOL	-5.43	-1.74	-3.59	3.69	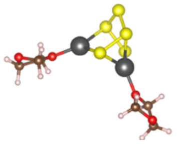
Li_2S_5-3DOL	-5.10	-1.54	-3.32	3.56	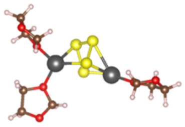
Li_2S_5-4DOL	-5.10	-1.60	-3.35	3.50	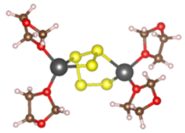
Li_2S_5-6DOL	-4.79	-1.42	-3.10	3.37	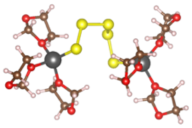
Li_2S_6	-5.76	-2.00	-3.88	3.76	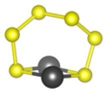
Li_2S_6-2DOL	-5.62	-1.92	-3.77	3.71	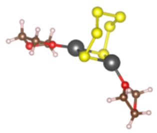
Li_2S_6-3DOL	-5.51	-1.83	-3.67	3.68	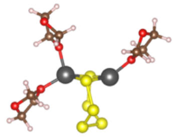
Li_2S_6-4DOL	-5.08	-1.91	-3.50	3.17	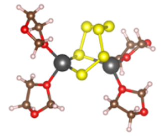
Li_2S_6-6DOL	-4.90	-1.70	-3.30	3.20	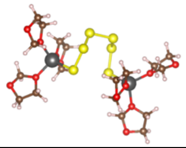
Li_2S_7	-5.75	-2.25	-4.00	3.50	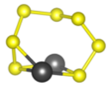
Li_2S_7-2DOL	-5.66	-2.05	-3.85	3.60	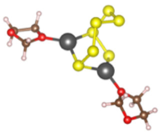

Li_2S_7-3DOL	-5.53	-2.02	-3.77	3.51	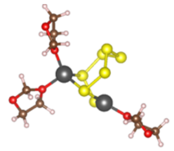
Li_2S_7-4DOL	-5.23	-2.11	-3.67	3.11	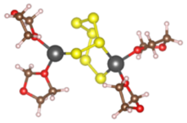
Li_2S_7-6DOL	-5.07	-1.77	-3.42	3.30	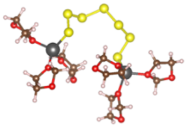
Li_2S_8	-5.80	-2.57	-4.18	3.22	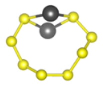
Li_2S_8-2DOL	-5.62	-2.54	-4.08	3.08	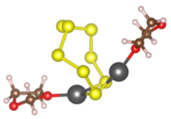
Li_2S_8-3DOL	-5.24	-2.46	-3.85	2.78	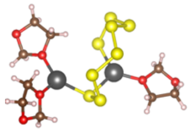
Li_2S_8-4DOL	-5.36	-2.45	-3.90	2.91	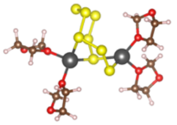
Li_2S_8-6DOL	-5.20	-2.07	-3.63	3.13	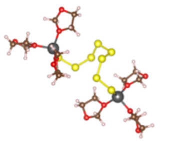

Supplementary Note 2: G-factors of sulfur radicals. We studied four LiS_y radicals, including LiS_5, LiS_4, LiS_3, LiS_2, and the effect of different explicit solvation molecules (DOL) on the g-factors. Firstly, the experimental measured *g*-factors in current work are g_{xx}=2.00421, g_{yy}=2.03770, g_{zz}=2.05694 (as shown in Fig. S1), which are close to the work of Vijayakumar *et al* (g_{xx}=2.0028, g_{yy}=2.0333, g_{zz}=2.0535)[1]. It should be notated that Vijayakumar's work uses a different solvent (DMSO). It is very difficult to reproduce the exact solvation environment in static DFT calculations because of the dynamic nature of solvation effect, which needs a long molecular dynamics simulation with expensive functionals. However, we can crudely estimate the influence of the solvent on measured g factors by comparing our results (in mixed DOL/DME) and the work of Vijayakumar (in DMSO), which present a difference in the level δ<0.01. Therefore, we can expect an error of the level δ~0.01 in DFT calculated g factors. In this work, we investigated solvated radicals by combing explicit solvent (DOL) and implicit solvent (PCM model). The results are shown in Table 3, in which basis sets of two different sizes are used. Apparently, LiS_3 radicals and LiS_4 radicals show much better agreement with experimental measured *g*-factors.

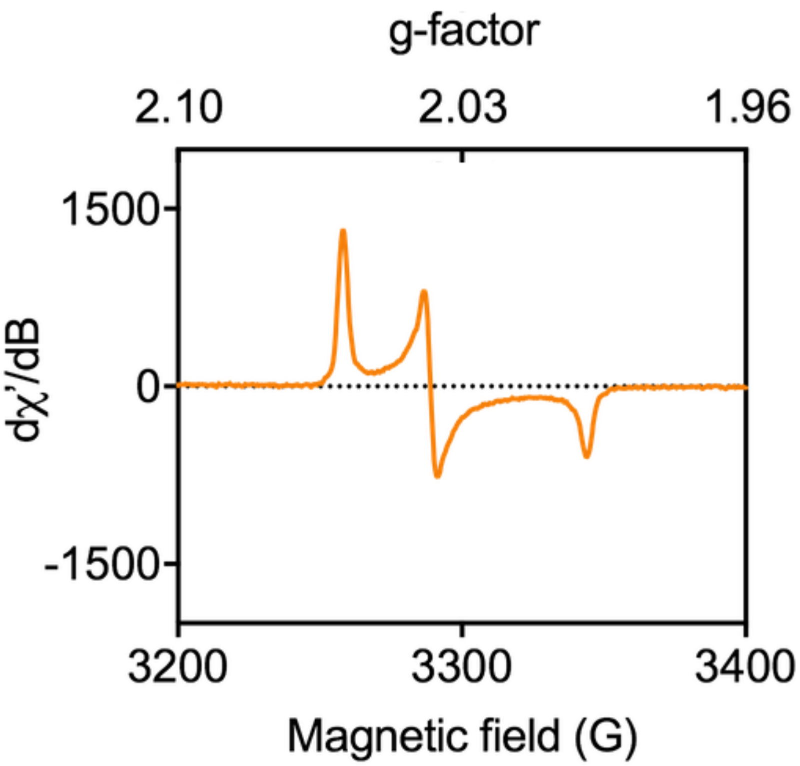

Supplementary Figure 1. EPR spectrum of 20 mM Li_2S_6 solution at 5 K, showing characteristic EPR spectra of LiS_3 radical. In an aprotic solvent, Li_2S_6 undergoes dissociation reactions and produces LiS_3 radicals, which is also detected in cycled Li–S cells.

Supplementary Table 2. Comparison of *g*-factors obtained from DFT simulation (G09 packages) and EPR measurement. Errors of the simulated *g*-factors for different structures of LiS_y radicals are shown. The errors are calculated with respect to the experimental values (g_{xx}=2.00421, g_{yy}=2.03770, g_{zz}=2.05694).

Radical formula	6-311++*g*(d,p)			cc-pvtz		
	g_{xx}	g_{yy}	g_{zz}	g_{xx}	g_{yy}	g_{zz}
LiS_2	-0.00242	-0.00193	0.11000	-0.00245	-0.00431	0.08898
LiS_2-1DOL	-0.00251	-0.00187	0.13194	-0.00253	-0.00427	0.10480
LiS_2-2DOL	-0.00255	-0.00184	0.17910	-0.00257	-0.00419	0.13926
LiS_2-3DOL	-0.00255	-0.00259	0.41262	-0.00259	-0.00474	0.28285
LiS_3	-0.00278	0.00348	-0.00472	-0.00278	0.00028	-0.00865
LiS_3-1DOL	-0.00281	0.00419	-0.00312	-0.00281	0.00085	-0.00724
LiS_3-2DOL	-0.00280	0.00552	-0.00001	-0.00280	0.00222	-0.00457
LiS_3-3DOL	-0.00280	0.00468	0.00305	-0.00282	0.00150	-0.00200
LiS_4	-0.00311	-0.00266	0.00554	-0.00315	-0.00450	0.00095
LiS_4-1DOL	-0.00309	-0.00236	0.00715	-0.00313	-0.00417	0.00231
LiS_4-2DOL	-0.00304	-0.00114	0.01345	-0.00311	-0.00308	0.00760
LiS_4-3DOL	-0.00284	-0.00532	0.03305	-0.00284	-0.00706	0.02354
LiS_5	0.00416	-0.01501	0.01855	0.00405	-0.01612	0.00943
LiS_5-1DOL	0.00491	-0.01544	0.02241	0.00469	-0.01642	0.01234
LiS_5-2DOL	0.00544	-0.01472	0.02132	0.00515	-0.01545	0.01260
LiS_5-3DOL	0.00493	-0.00787	0.03794	0.00401	-0.00917	0.02458

Supplementary Table 3. Geometric and electronic band structures of LiS_m radicals with different solvation models. HOMO, LUMO, Band gap center (BGC) and Band gap (LUMO-HOMO) of Li_2S_x molecules. The calculations are conducted with Gaussian09 package using 6-311++G(d,p) basis set and B3LYP functional. Environmental solvents are considered with implicit solvation model ($\varepsilon = 4.33$). The band gap center (BGC) is the average of the HOMO and LUMO levels.

	HOMO (eV)	LUMO (eV)	BGC (eV)	Band gap (eV)	Structure
LiS_2	-5.36	-3.29	-4.32	2.07	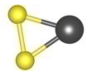
LiS_2-1DOL	-5.27	-3.22	-4.24	2.05	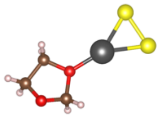
LiS_2-2DOL	-5.12	-3.12	-4.12	2.00	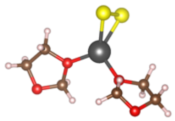
LiS_2-3DOL	-4.70	-2.90	-3.80	1.81	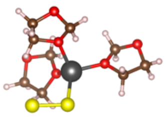
LiS_3	-5.65	-3.58	-4.61	2.07	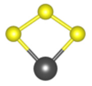
LiS_3-1DOL	-5.57	-3.50	-4.53	2.07	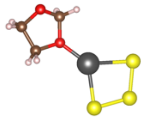
LiS_3-2DOL	-5.45	-3.38	-4.42	2.07	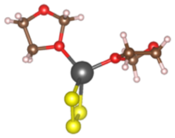
LiS_3-3DOL	-5.18	-3.12	-4.15	2.07	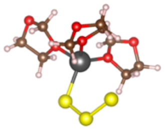

LiS_4	-5.48	-3.68	-4.58	1.80	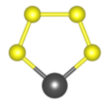
LiS_4-1DOL	-5.41	-3.62	-4.52	1.79	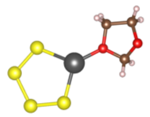
LiS_4-2DOL	-5.37	-3.59	-4.48	1.78	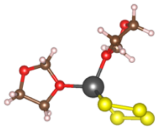
LiS_4-3DOL	-5.31	-3.51	-4.41	1.80	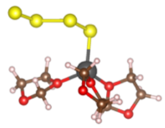
LiS_5	-5.91	-4.11	-5.01	1.80	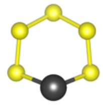
LiS_5-1DOL	-5.90	-4.12	-5.01	1.78	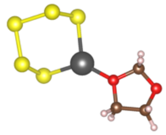
LiS_5-2DOL	-5.70	-3.98	-4.84	1.72	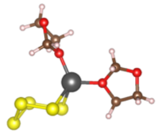
LiS_5-3DOL	-5.34	-3.97	-4.66	1.38	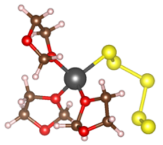

Supplementary Note 3: Calculations on Li_2S_2. In this work, we exploited a Li_2S_2 structure proposed by Yang et al.[2], which is determined by global optimization method. This Li_2S_2 structure adopts a tetragonal cell with the symmetry as *P_{42}/mnm*. The calculated band gap is 2.29 eV, which is slightly larger than previous DFT predicted value of 1.8 eV[2] and the discrepancy may blame to the different functionals (PBE vs SCAN in this work). Park et al. studied a different Li_2S_2 model (symmetry *P_{63}/mmc*) and predicted a band gap with 2.70 eV with GGA+G_0W_0[3]. One can conclude from those results that the persulfide bond (S-S) bond is a common feature in the meta-stable Li_2S_2 phases and Li_2S_2 is supposed to be an insulator with a relatively large band gap (~2 eV). Though, current Li_2S_2 structure is different from the structures proposed by Feng et al[4]. and other previous papers that contains S_2^{2-} anions and the LiS_4 tetrahedrons. These structures are supposed to exhibit similar chemical properties.

Supplementary Note 4: Calculations on orthorhombic Nb_2O_5/$LiNb_2O_5$. The experimental unit cell of T-phase Nb_2O_5 contains 16.8 niobium cations and 42 oxygen anions[5]. Given that a theoretical model which fulfills both the experimental fractional occupations and stoichiometry is too large for the first-principles calculations, we followed the procedure proposed by Pinto[6] to build a moderate size Nb_2O_5 (formula is $Nb_{16}O_{40}$). As shown in the work of Pinto et al, this simplified model does not compromise the accuracy. In terms of the locations of inserted lithium cations, we exploited a molecular dynamic (MD) simulation (with T=1000 K and 3000 steps) to generate promising configurations and then the low energy frames in the MD trajectory are further optimized to reduce the maximum force residue below 0.01 eV/Ang. The lattice constants before/after the lithiation are listed in Table 6.

Supplementary Table 4. Lattice parameters of T-Nb_2O_5 and $LiNb_2O_5$.

x in $Li_xNb_2O_5$	a	b	c	α	β	γ
0.0	6.176	29.175	3.931	90	90	90
1.0	6.315	29.117	3.931	90.006	90.064	90.617

Supplementary Note 5: Calculations on birnessite MnO_2/$Li_{0.5}MnO_2$: The layered structure of MnO_2 is adapted from Gaillot's work[7]. In this study we are using a 2×2×1 supercell (formula is Mn_4O_8) as the unit cell due to the requirement of the antiferromagnetic spin arrangement of MnO_2 crystals. The large supercell also enables us to study different lithiation structures. The unit cell is in hexagonal structure and shows symmetry of *P_{63}/mmc*. All the Mn^{4+} cations exist as MnO_6 octahedra and share edges in the same layer. The interlayer distance is variable according to the number of inserted water molecules or cations. The optimized MnO_2 in this work shows small interlayer distance 4.75 Å, which is smaller than the experimental potassium-contained Birnessite[7,8]. The Mn-O bond distance is 1.890 Å and Mn-Mn distance (in the same layer) is 2.827 Å. The results agree well with other computations[9] and does not change a lot compared to potassium Birnessite[7]. The calculated band gap of MnO_2 in this work is 1.4 eV (see DOS in Fig.

2), which is consistent with the work of Kwon[10], but smaller than the experimental reported value 2.23 eV[11].

Supplementary Table 5. Lattice Parameters of MnO_2 and $Li_{0.5}MnO_2$.

x in Li_xMnO_2	a	b	c	α	β	γ
0.0	5.654	5.654	9.506	90.0	90.0	120.04
0.5	5.661	5.691	9.651	89.8	101.4	118.08

Supplementary Table 6. Band edge positions of Li_2S_2, $LiNb_2O_5$, Nb_2O_5, $Li_{0.5}MnO_2$ and MnO_2 with SCAN and HSE06 functionals. Although the hybrid functional (HSE06) shows larger band gaps than meta-GGA (SCAN) functional, the derived BGCs reasonably agree. Li_sv(3e) indicates the pseudopotential of Li includes the 1s electrons, and the energy cutoffs for those calculations were 520 eV.

	Band gap (eV)	VBM (eV)	CBM (eV)	BGC (eV)
SCAN functional				
Li_2S_2 (Li sv (3e), 520)	2.28	-4.94	-2.65	-3.80
$LiNb_2O_5$ (Li sv (3e), 520)	0.00 (metallic)	-4.17	-4.17	-4.17
$LiNb_2O_5$ (Li (1e), 400)	0.00 (metallic)	-4.13	-4.13	-4.13
Nb_2O_5 (400)	2.36	-5.96	-3.60	-4.78
$Li_{0.5}MnO_2$ (Li sv (3e), 520)	0.00 (metallic)	-7.10	-7.10	-7.10
$Li_{0.5}MnO_2$ (Li (1e), 400)	0.00 (metallic)	-6.86	-6.86	-6.86
MnO_2 (400)	1.20	-8.03	-6.83	-7.43
HSE06 functional				
Li_2S_2 (HSE06)	3.04	-4.98	-1.95	-3.47
MnO_2 (HSE06)	2.95	-9.15	-6.20	-7.67
Nb_2O_5 (HSE06)	2.55	-6.36	-3.81	-5.08
$LiNb_2O_5$ (HSE06)	0.0 (metallic)	-4.08	-4.08	-4.08

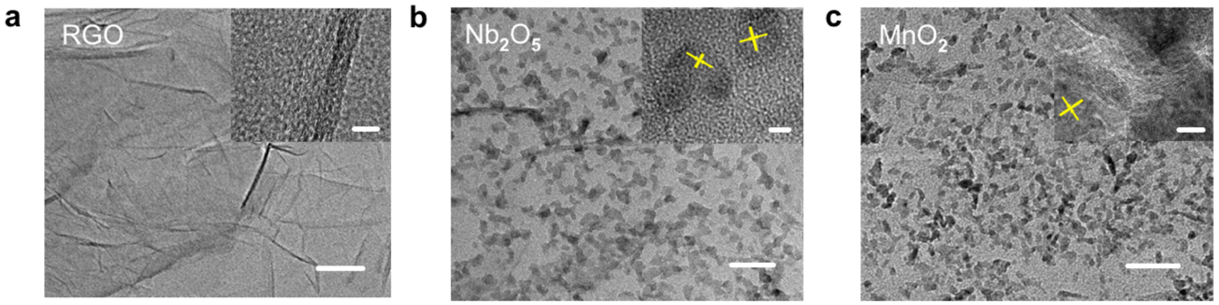

Supplementary Figure 2. TEM images and high resolution TEM images (inset) of **a**, RGO, **b**, Nb_2O_5, **c**, MnO_2 composites; Scale bars are 50 nm in TEM, and 5 nm in HRTEM. The *d*-spacings of the Nb_2O_5 and MnO_2 nanoparticles are 0.395 nm and 0.24 nm, correspond to (001) lattice plane of orthorhombic Nb_2O_5 and (211) lattice plane of birnessite MnO_2, respectively.

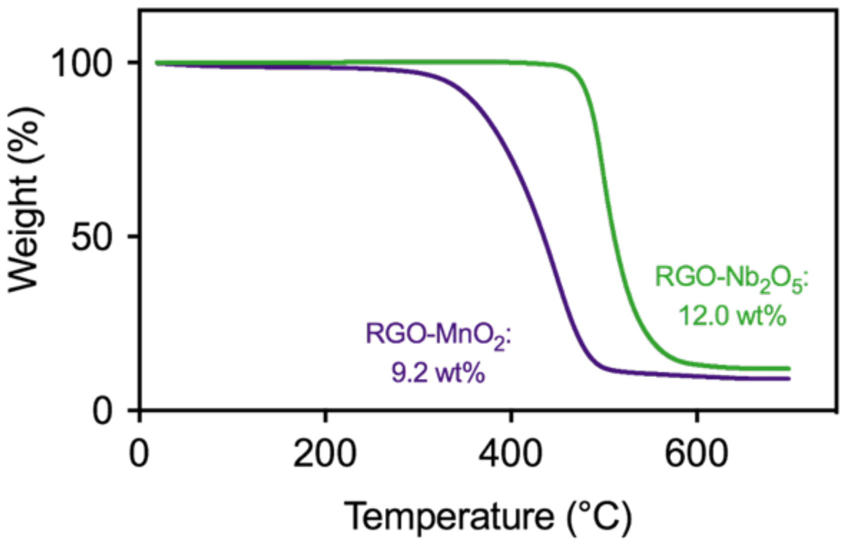

Supplementary Figure 3. Thermogravimetric analysis of RGO-based composites.

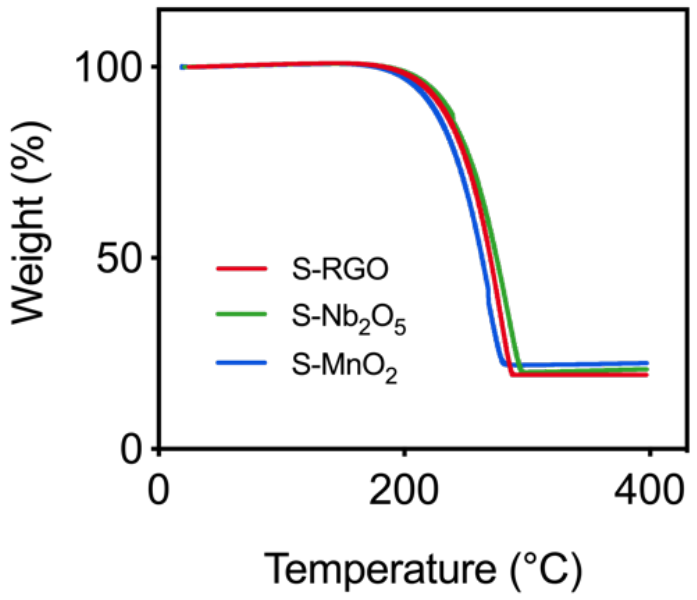

Supplementary Figure 4. Thermogravimetric analysis of S–RGO, S–RGO–Nb_2O_5, and S–RGO–MnO_2 composites. The weight percentage of sulfur is ~ 80 wt% in these composites.

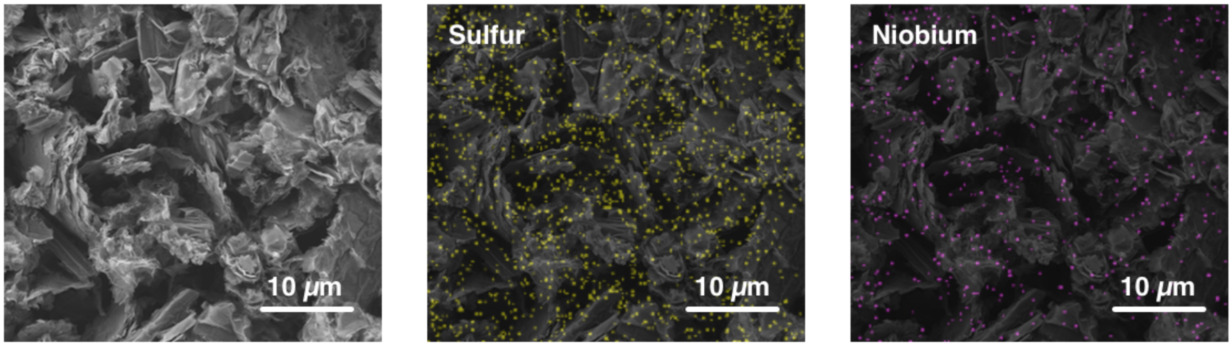

Supplementary Figure 5. SEM images of electrode based on S-Nb_2O_5 composites.

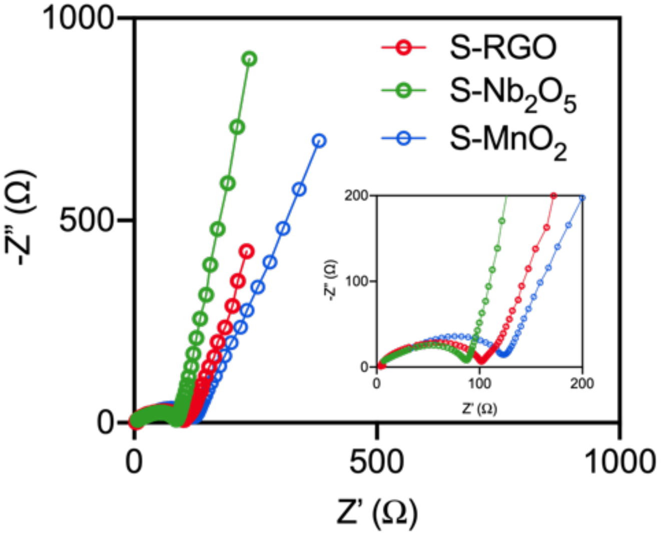

Supplementary Figure 6. Initial EIS of Li-S cells with different sulfur electrodes, showing similar charge transfer resistances.

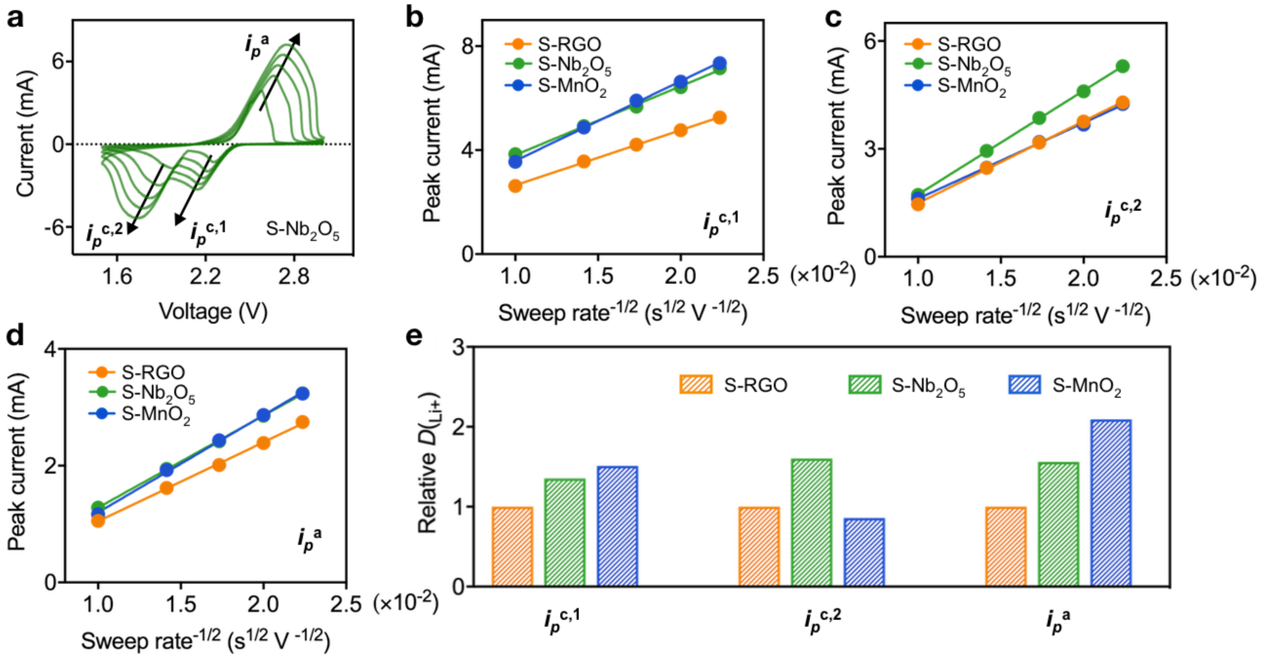

Supplementary Figure 7. Electrochemical performance of sulfur cathodes with/without redox mediators. a. Cyclic voltammetry of a S–Nb_2O_5 electrode under various scanning rates, ranging from 0.1 mV s^{-1} to 0.5 mV s^{-1}. **b–d.** Plots of cathodic and anodic peak currents *vs.* square root of sweep rates. The linear relationship between I_p and $v^{0.5}$ indicates the electrochemical behavior of sulfur cathodes is a diffusion–limited process. **e.** Relative lithium ion diffusion coefficient $D(Li^+)$ of different electrochemical steps.

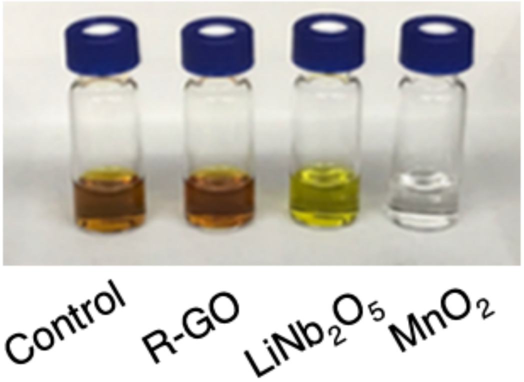

Supplementary Figure 8. Photographs of Li_2S_6 solutions mixed with RGO, $LiNb_2O_5$ and MnO_2 after centrifugation.

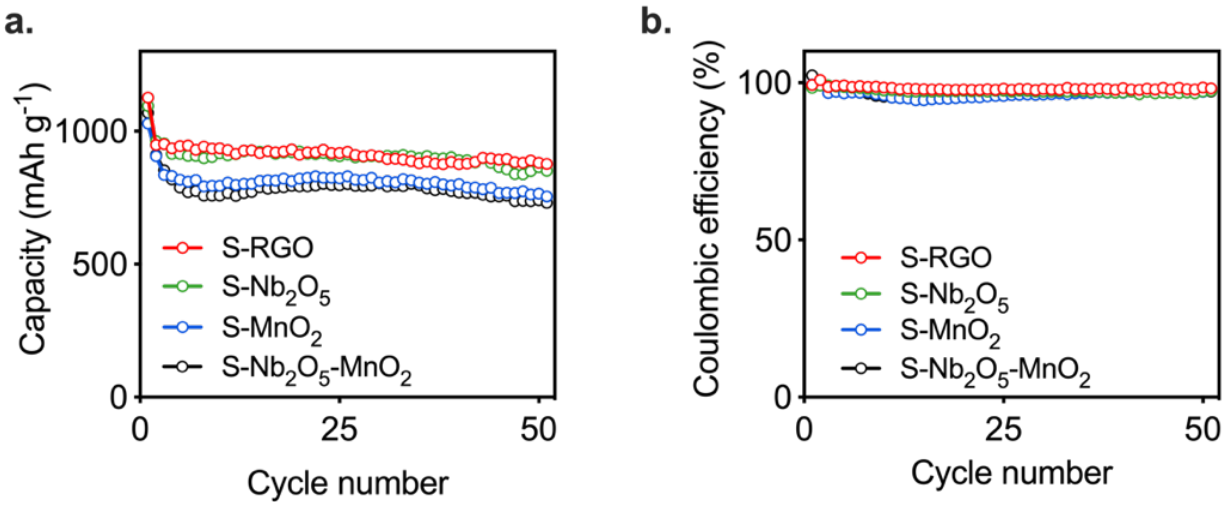

Supplementary Figure 9. a, Specific capacity and **b,** Coulombic efficiency of thin sulfur electrodes (sulfur mass loading 1.5 - 2 mg cm^{-2}) with different oxides. All the cells were first activated at 0.1 C rate for the first cycle. During galvanostatic cycling, cells were charged at 0.3 C rate (1 C rate corresponds to 1675 mA g^{-1}) and subsequently discharged at 0.1 C rate. For thin sulfur electrodes, the improvement can be marginal or even negligible.

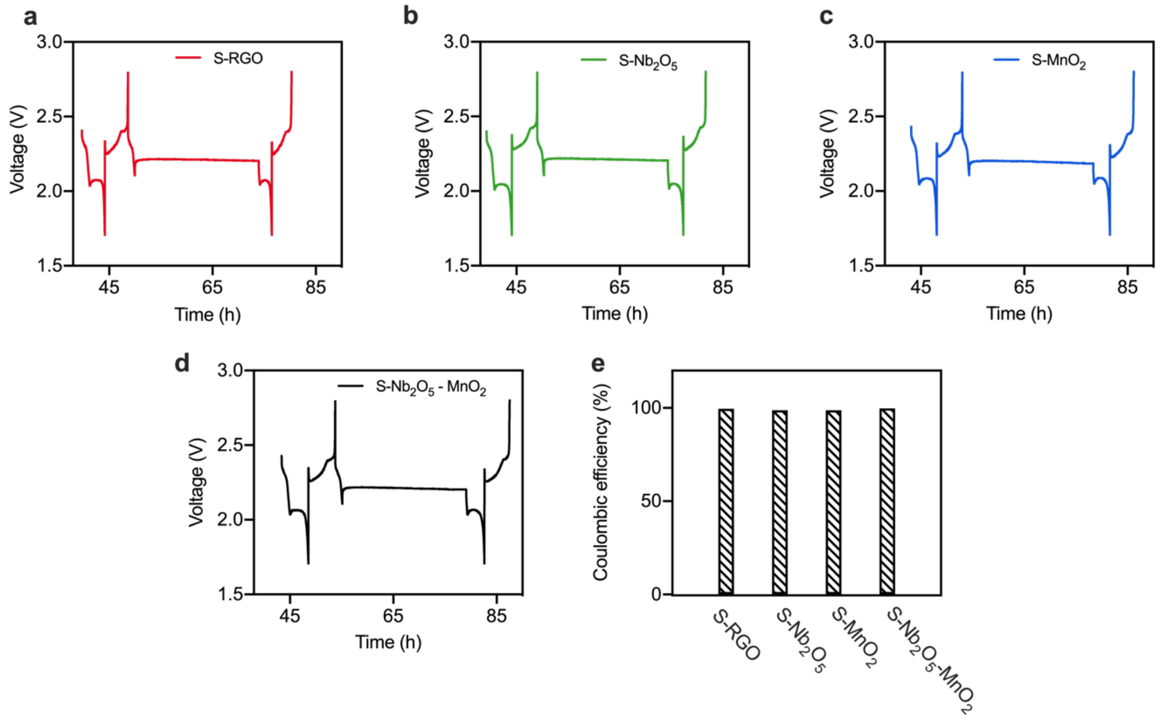

Supplementary Figure 10. Voltage profile of **a.** S-RGO, **b.** S-Nb_2O_5, **c.** S-MnO_2 and **d.** S-Nb_2O_5-MnO_2 during self-discharging test and their Coulombic efficiency during the 5th cycle (resting cycle).

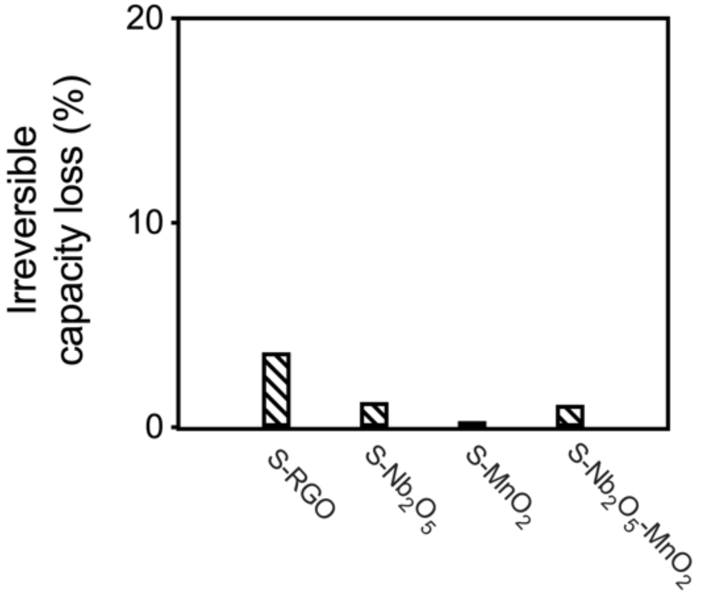

Supplementary Figure 11. Irreversible capacity loss of S-RGO, S-Nb_2O_5, S-MnO_2, S-Nb_2O_5-MnO_2 electrodes after resting for 24 hours at 2.1 V (*vs.* Li^+/Li) during the 5th discharging step. The irreversible capacity loss is defined as $(C_4\text{-}C_6)/C_4 \times 100\%$ (C_n represent the discharge capacity during the nth cycle).

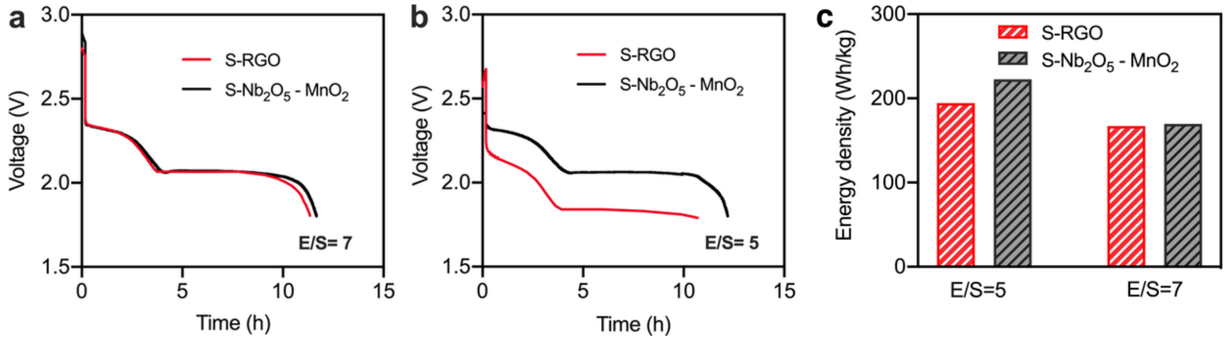

Supplementary Figure 12. Initial discharge voltage profile of S-RGO and S-Nb_2O_5-MnO_2 electrodes at 0.05 C rate with different amount of electrolyte **a.** E/S = 7 and **b.** E/S = 5. **c.** Energy density of Li-S cells with different E/S ratios.

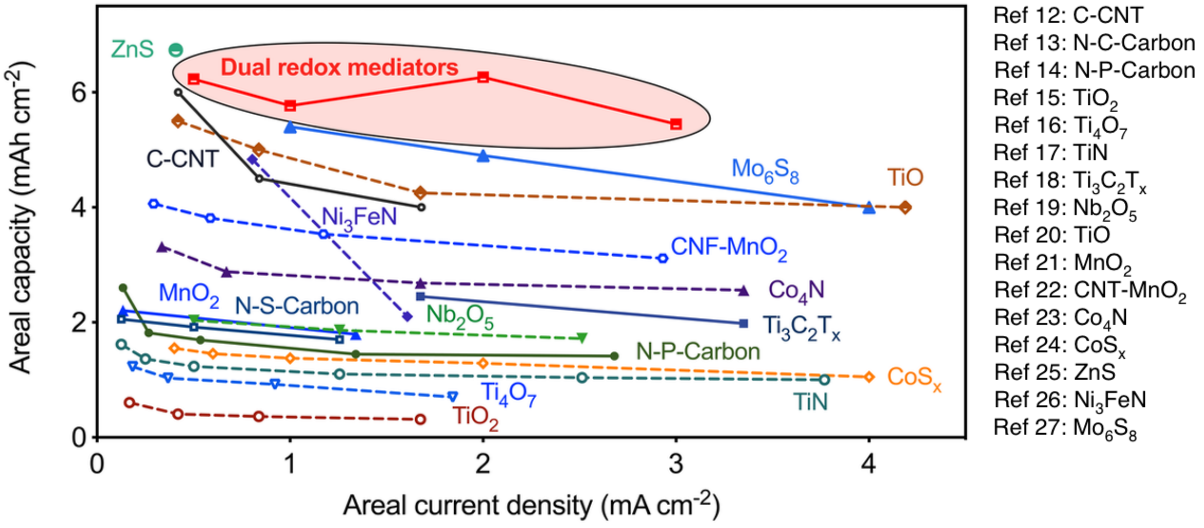

Supplementary Figure 13. Areal capacity and areal current densities of S-Nb_2O_5-MnO_2 and S-RGO electrodes. Cells with other representative catalytic agents are also plotted for comparison[12-27].

References

1. Vijayakumar, M. *et al.* Molecular structure and stability of dissolved lithium polysulfide species. *Phys. Chem. Chem. Phys.* **16,** 10923–10932 (2014).
2. Yang, G., Shi, S., Yang, J. & Ma, Y. Insight into the role of Li_2S_2 in Li–S batteries: a first-principles study. *J. Mater. Chem. A* **3,** 8865–8869 (2015).
3. Park, H., Koh, H. S. & Siegel, D. J. First-principles study of redox end members in lithium–sulfur batteries. *J. Phys. Chem. C* **119,** 4675–4683 (2015).
4. Feng, Z. *et al.* Unravelling the role of Li_2S_2 in lithium–sulfur batteries: A first principles study of its energetic and electronic properties. *J. Power Sources* **272,** 518–521 (2014).
5. Von Tamura, K. Die Kristallstruktur von T-Nb_2O_5.
6. Pinto, M. B., Soares, A. L., Mella Orellana, A., Duarte, H. A. & De Abreu, H. A. Structural, electronic, and thermodynamic properties of the T and B phases of niobia: First-principle calculations. *J. Phys. Chem. A* **121,** 2399–2409 (2017).
7. Gaillot, A.-C. *et al.* Structure of synthetic K-rich Birnessite obtained by high-temperature decomposition of $KMnO_4$. I. Two-layer polytype from 800 °C experiment. *Chem. Mater.* **15,** 4666–4678 (2003).
8. Yeager, M. *et al.* Highly efficient $K_{0.15}MnO_2$ birnessite nanosheets for stable pseudocapacitive cathodes. *J. Phys. Chem. C* **116,** 20173–20181 (2012).
9. Kwon, K. D., Refson, K. & Sposito, G. On the role of Mn(IV) vacancies in the photoreductive dissolution of hexagonal birnessite. *Geochim. Cosmochim. Acta* **73,** 4142–4150 (2009).
10. Kwon, K. D., Refson, K. & Sposito, G. Defect-induced photoconductivity in layered manganese oxides: a density functional theory study. *Phys. Rev. Lett.* **100,** 146601 (2008).
11. Sakai, N., Ebina, Y., Takada, K. & Sasaki, T. Photocurrent generation from semiconducting manganese oxide nanosheets in response to visible light. *J. Phys. Chem. B* **109,** 9651–9655 (2005).
12. Xu, T. *et al.* Mesoporous carbon-carbon nanotube-sulfur composite microspheres for high-areal-capacity lithium-sulfur battery cathodes. *ACS Appl. Mater. Interfaces* **5,** 11355–11362 (2013).
13. Wu, H. *et al.* A high-efficiency N/P co-doped graphene/CNT@porous carbon hybrid matrix as a cathode host for high performance lithium–sulfur batteries. *J. Mater. Chem. A* **5,** 20458–20472 (2017).
14. Pang, Q. *et al.* A nitrogen and sulfur dual-doped carbon derived from polyrhodanine@cellulose for advanced lithium-sulfur batteries. *Adv. Mater.* **27,** 6021–6028 (2015).
15. Wei Seh, Z. *et al.* Sulphur-TiO_2 yolk-shell nanoarchitecture with internal void space for long-cycle lithium-sulphur batteries. *Nat. Commun.* **4,** 1331 (2013).
16. Mei, S. *et al.* Porous Ti_4O_7 particles with interconnected-pore structure as a high-efficiency polysulfide mediator for lithium-sulfur batteries. *Adv. Funct. Mater.* **27,** 1701176 (2017).
17. Hao, Z. *et al.* TiN as a simple and efficient polysulfide immobilizer for lithium–sulfur batteries. *J. Mater. Chem. A* **4,** 17711–17717 (2016).
18. Bao, W., Su, D., Zhang, W., Guo, X. & Wang, G. 3D metal carbide@mesoporous carbon hybrid architecture as a new polysulfide reservoir for lithium-sulfur batteries. *Adv. Funct. Mater.* **26,** 8746–8756 (2016).
19. Tao, Y. *et al.* Kinetically-enhanced polysulfide redox reactions by Nb_2O_5 nanocrystals for high-rate lithium–sulfur battery. *Energy Environ. Sci.* **9,** 3230–3239 (2016).
20. Li, Z., Guan, B. Y., Zhang, J. & Lou, X. W. (David). A compact nanoconfined sulfur cathode for high-performance lithium-sulfur batteries. *Joule* **1,** 576–587 (2017).
21. Liang, X. & Nazar, L. F. In situ reactive assembly of scalable core-shell sulfur-MnO_2

composite cathodes. *ACS Nano* **10,** 4192–4198 (2016).
22. Li, Z., Zhang, J. & Lou, X. W. D. Hollow carbon nanofibers filled with MnO_2 nanosheets as efficient sulfur hosts for lithium-sulfur batteries. *Angew. Chem. Int. Ed.* **54,** 12886–12890 (2015).
23. Deng, D.-R. *et al.* Co_4N nanosheet assembled mesoporous sphere as a matrix for ultrahigh sulfur content lithium-sulfur batteries. *ACS Nano* **11,** 6031–6039 (2017).
24. Lao, M. *et al.* Homogeneous sulfur–cobalt sulfide nanocomposites as lithium–sulfur battery cathodes with enhanced reaction kinetics. *ACS Appl. Energy Mater.* **1**, 167-172 (2018).
25. Shin, W., Lu, J. & Ji, X. ZnS coating of cathode facilitates lean-electrolyte Li-S batteries. *Carbon Energy* **1,** 165–172 (2019).
26. Zhao, M. *et al.* Activating inert metallic compounds for high-rate lithium-sulfur batteries through in situ etching of extrinsic metal. *Angew. Chem. Int. Ed.* **58,** 3779–3783 (2019).
27. Xue, W. *et al.* Intercalation-conversion hybrid cathodes enabling Li–S full-cell architectures with jointly superior gravimetric and volumetric energy densities. *Nat. Energy* **4,** (2019).
